# Identification of non-ischaemic fibrosis in male veteran endurance athletes, mechanisms and association with premature ventricular beats

**DOI:** 10.1038/s41598-023-40252-z

**Published:** 2023-09-05

**Authors:** Maryum Farooq, Louise A. E. Brown, Andrew Fitzpatrick, David A. Broadbent, Ali Wahab, Joel R. L. Klassen, Jonathan Farley, Christopher E. D. Saunderson, Arka Das, Thomas Craven, Erica Dall’Armellina, Eylem Levelt, Hui Xue, Peter Kellman, John P. Greenwood, Sven Plein, Peter P. Swoboda

**Affiliations:** 1 Biomedical Imaging Sciences Department, Leeds Institute of Cardiovascular and Metabolic Medicine, University of Leeds, Leeds, LS2 9JT UK; 2 Cardiac Investigations Unit, Leeds Teaching Hospitals NHS Trust, Leeds, UK; 3 Medical Physics and Engineering, Leeds Teaching Hospitals NHS Trust, Leeds, UK; 4 National Heart, Lung, and Blood Institute, National Institutes of Health, DHHS, Bethesda, MD USA

**Keywords:** Cardiology, Medical research

## Abstract

Left ventricular fibrosis can be identified by late gadolinium enhancement (LGE) cardiovascular magnetic resonance (CMR) in some veteran athletes. We aimed to investigate prevalence of ventricular fibrosis in veteran athletes and associations with cardiac arrhythmia. 50 asymptomatic male endurance athletes were recruited. They underwent CMR imaging including volumetric analysis, bright blood (BB) and dark blood (DB) LGE, motion corrected (MOCO) quantitative stress and rest perfusion and T1/T2/extracellular volume mapping. Athletes underwent 12-lead electrocardiogram (ECG) and 24-h ECG. Myocardial fibrosis was identified in 24/50 (48%) athletes. All fibrosis was mid-myocardial in the basal-lateral left ventricular wall. Blood pressure was reduced in athletes without fibrosis compared to controls, but not athletes with fibrosis. Fibrotic areas had longer T2 time (44 ± 4 vs. 40 ± 2 ms, *p* < 0.0001) and lower rest myocardial blood flow (MBF, 0.5 ± 0.1 vs. 0.6 ± 0.1 ml/g/min, *p* < 0.0001). On 24-h ECG, athletes with fibrosis had greater burden of premature ventricular beats (0.3 ± 0.6 vs. 0.05 ± 0.2%, *p* = 0.03), with higher prevalence of ventricular couplets and triplets (33 vs. 8%, *p* = 0.02). In veteran endurance athletes, myocardial fibrosis is common and associated with an increased burden of ventricular ectopy. Possible mechanisms include inflammation and blood pressure. Further studies are needed to establish whether fibrosis increases risk of malignant arrhythmic events.

## Introduction

Sedentary lifestyle leads to shortened life expectancy and increases cardiovascular risk. Even moderate amounts of exercise can reduce cardiovascular risk via improvements in blood pressure, lipid profile and insulin resistance^1,2^. Most developed countries recommend a minimum weekly amount of moderate intensity exercise. The British Association of Sports and Exercise Sciences recommends a minimum of 150 min moderate intensity or 75 min high intensity exercise per week^3^.

Habitual moderate intensity exercise improves all cause and cardiovascular mortality, but it remains unclear if these benefits extend to those who participate in high intensity exercise into later life. Whilst some studies have shown lower mortality in elite endurance athletes^4^, others suggesting a loss of benefit have received significant attention from the popular media^5^.

Several studies have reported myocardial fibrosis detected by late gadolinium enhancement (LGE) cardiovascular magnetic resonance (CMR) in veteran athletes although both pattern and prevalence (4–14%) have varied^6–8^. The presence of non-ischaemic fibrosis is associated with adverse outcomes including ventricular arrhythmia in a variety of conditions including dilated, hypertrophic and arrhythmogenic cardiomyopathies^9^. It is unknown whether non-ischaemic fibrosis is associated with adverse outcomes in healthy lifelong athletes.

The aim of this study was to evaluate the prevalence of myocardial fibrosis in male veteran athletes. We also aimed to evaluate associated clinical and sporting factors and if fibrosis was associated with abnormalities on the 12-lead electrocardiogram (ECG) and 24-h cardiac monitor.

## Methods

All methods were performed in accordance with the relevant guidelines and regulations.

### Participants

Athletes were recruited through advertisements at local cycling and triathlon clubs. Male athletes aged fifty and above who undertook > ten hours of training a week for > fifteen years and competed regularly were recruited. Age matched male control subjects who trained < 3 h per week were also recruited. Exclusion criteria for all participants included prior cardiovascular disease, pre-existing diabetes or hypercholesterolaemia, use of cardiac medications, smoking and contraindication to CMR. The study was approved by the University of Leeds Research Ethics Committee and all participants gave written informed consent.

In a single visit, athletes underwent clinical assessment, contrast enhanced CMR, 12-lead and twenty-four-hour ECG monitoring. Clinical assessment included body composition analysis (RD-545, Tanita, Tokyo, Japan). Both 12-lead ECG (MAC500, GE Medical Systems, Milwaukee, WI, USA) and 24-h ECG monitoring (Lifecard CF holters, Spacelabs Healthcare, Washington, USA) analyses were blinded to clinical details^10^. Participants were also asked to report their lifetime competition history using a standardised questionnaire.

### Cardiovascular magnetic resonance protocol

Participants underwent CMR on a 3.0 Tesla system (Prisma, Siemens Healthineers, Erlangen, Germany). All participants abstained from caffeine for twenty-four hours prior to the scan. A full blood count, for measurement of haematocrit, was taken at the time of intravenous cannulation prior to each CMR study.

Left ventricle function (cine) imaging was performed in standard long and short axis planes using a fast gradient echo sequence prior to contrast administration (10–12 slices, slice thickness 10 mm with no gap, 25 cardiac phases).

Mapping and perfusion imaging were acquired in four short axis slices planned at end-systole (apex, mid, base and outflow levels). Imaging at the outflow level was included as this is a common site for fibrosis in athletes^11^. Native T1 mapping used a breath-held 5 s(3 s)3 s Modified Look-Locker Inversion recovery (MOLLI) acquisition.

Hyperaemia was induced using an intravenous adenosine infusion at 140-210mcg/kg/min with dose increased if no symptomatic or haemodynamic response. Perfusion imaging was acquired using a previously validated automated method incorporating in-line motion correction and myocardial blood flow (MBF) quantification^12^. Data were acquired in four slices with the arterial input function measured from the basal slice to avoid outflow tract. Rest perfusion images were acquired once the effects of adenosine had subsided, at least fifteen minutes after the final stress perfusion sequence. Intravenous gadobutrol (Gadovist®, Bayer Pharma, Berlin, Germany) was administered at a dose of 0.05 mmol/kg for each of stress, rest and top up.

Bright blood (BB) late gadolinium enhancement (LGE) was carried out six minutes after final contrast administration using a T1-weighted, segmented inversion-recovery sequence in multiple planes (free-breathing with MOCO). A further short axis stack of dark blood (DB) images with complete LV coverage were acquired^13^.

Post contrast T1 mapping was carried out > 15 min after final contrast injection using 4 s(1 s)3 s(1 s)2 s MOLLI acquisition with positioning and planning as per the native T1 map.

### Cardiovascular magnetic resonance analysis

**Figure 1:**
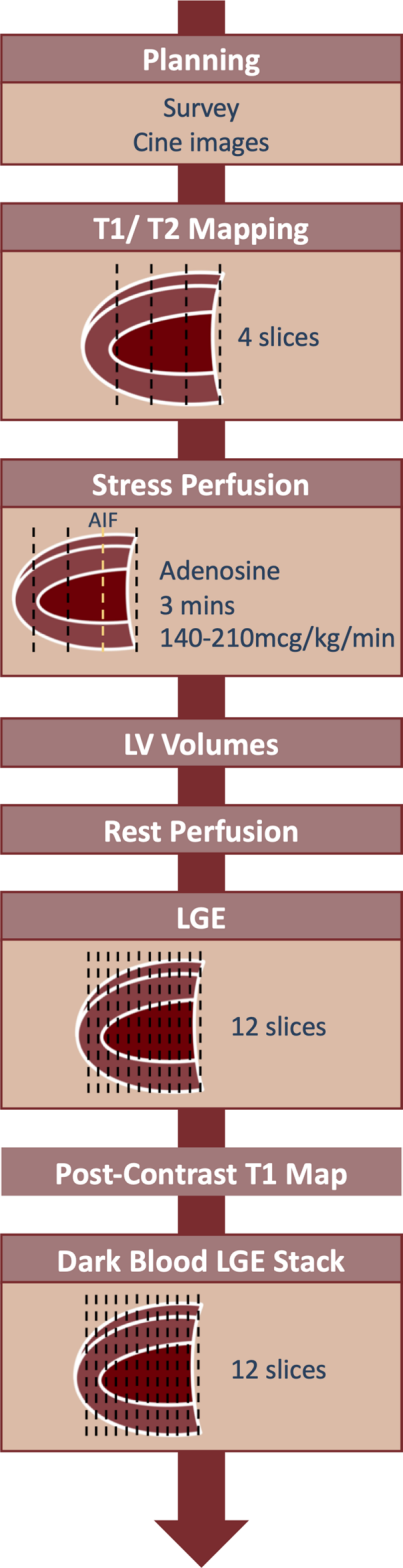
CMR Protocol. Participants underwent CMR on a 3.0 Tesla Siemens Prisma system. The protocol consisted of cine, T1/T2 mapping, stress imaging using intravenous adenosine infusion, bright and dark blood imaging. LV = Left ventricle. LGE = Late gadolinium enhancement.

The CMR data was analysed using cvi42 software (Circle Cardiovascular Imaging Inc. Calgary, Canada). The protocol is summarised in Fig. 1. Epicardial and endocardial borders were drawn on short axis cine stacks to calculate LV mass, LV and RV end-diastolic volume (EDV), end systolic volume (ESV), stroke volume (SV) and ejection fraction (EF). Parameters were indexed to body surface area, calculated using the Mosteller equation.

The presence of fibrosis was assessed blinded to other results and was only included if it could be detected on at least two of BB LGE, DB LGE or ECV mapping. Myocardial fibrosis was quantified on short axis LGE images using the full width at half maximum (FWHM) technique.

Pre and post-contrast myocardial T1 values were measured from the basal and mid-ventricular short axis whole slices using 3-parameter exponential fit with Look-Locker correction. ECV was calculated using haematocrit, native and post-contrast T1 times for myocardium and blood pool as previously described^14^. Global T1, T2, ECV and MBF were measured from the basal and mid-ventricular short axis slices excluding areas of fibrosis. They were also measured in fibrotic and remote tissue by drawing regions of interest planned from LGE imaging.

For global measures apical and outflow tract slices were not included to avoid partial volume effects in thin myocardium. After exclusion of enhancing myocardium intracellular and extracellular compartment volume was calculated using equations previously reported^14^.

### Statistical analysis

Statistical analysis was performed using SPSS 23.0 (IBM SPSS, Armonk, NY, USA). The Shapiro–Wilk test was used to assess for normal distribution. Continuous variables are expressed as mean ± SD if normally distributed, or median and interquartile range if non-normally distributed. Categorical variables are expressed as N (%). Continuous data were compared using Student’s t test, and categorical using chi-squared test. *p* < 0.05 was considered statistically significant.

### Ethical approval

The study was approved by the University of Leeds Research Ethics Committee and all participants gave written informed consent.

### Consent

Informed consent has been obtained for publication of identifying images in an open-access publications.

## Results

### Demographics and exercise history

Fifty male athletes were recruited between August and December 2018, with a median age of 56 (IQR: 53–64) years. Forty-one of the participants were cyclists (average 662 competitions spanning 26 years) and nine were triathletes (average 171 competitions spanning 25 years). Participant characteristics are shown in Table 1.

**Table 1:** Clinical characteristics of control group and athletes according to the presence of fibrosis.

Characteristic	Healthy control	Athlete LGE-	Athlete LGE +	P value Control vs LGE- athlete	*P* value control versus LGE + athlete	*P* value LGE- versus LGE + athlete
N	26	26	24	–	–	–
Age	61.6 ± 7.6	56.8 ± 6.4	61.4 ± 8.0	0.067	1.0	0.10
Weight (kg)	82.6 ± 9.9	73.7 ± 6.9	72.9 ± 8.6	**0.001**	** < 0.001**	1.0
Height (cm)	174.8 ± 6.9	175.3 ± 4.2	172.3 ± 6.1	1.0	0.42	0.23
Body fat (%)	24.9 ± 6.2	16.8 ± 4.3	17.8 ± 3.8	** < 0.001**	** < 0.001**	1.0
Lean muscle mass (kg)	59.5 ± 5.2	57.8 ± 4.1	56.6 ± 6.3	0.98	0.32	1.0
Systolic blood pressure (mmHg)	129.4 ± 17.8	113.4 ± 22.9	123.2 ± 13.4	**0.008**	0.72	0.20
Diastolic blood pressure (mmHg)	78.5 ± 7.1	70.2 ± 7.5	75.1 ± 8.0	** < 0.001**	0.35	0.07
Heart rate	64.0 ± 11.3	54.5 ± 9.8	53.1 ± 7.1	**0.002**	** < 0.001**	1.0
Training volume (hours per week)	–	11.4 ± 1.8	11.4 ± 2.0	–	–	1.0
Cumulative training (years)	–	24.7 ± 10.8	27.7 ± 14.1	–	–	0.40
Discipline:						
Cycling	–	20	21	–	–	–
Triathlon	–	6	3	–	–	–

Values are mean ± standard deviation.

LGE- = Fibrosis absent; LGE +  = Fibrosis present.

Significant values are in [bold].

### Presence and extent of myocardial fibrosis

LGE was detected in 24/50 (48%) participants compared with only 4/26 (15%) matched controls (*p* = 0.005). In all athletes, the fibrosis was non-ischaemic, located in the mid-myocardium of the basal lateral wall of the left ventricle. Mean fibrosis volume in athletes was 3 ± 4 ml.

### Clinical and CMR differences between athletes with and without fibrosis

There was no significant difference in age between athletes with fibrosis (LGE +) and athletes without fibrosis (LGE-). The control group had a higher weight and body fat percentage compared to LGE + and LGE- athletes. There were no differences in blood pressure between athletes with and without fibrosis, but LGE- athletes had significantly lower systolic and diastolic blood pressure than controls (113/70 vs 129/79 mmHg). This pattern was not seen in LGE + athletes. Participant characteristics according to presence of fibrosis are shown in Table 1.

The control group had lower left ventricular mass, left and right ventricular end-diastolic volumes, and higher left ventricular ejection fraction. LGE + and LGE- athletes had comparable LV mass, left and right ventricular end-diastolic volumes and ejection fractions. There was no difference in rest or stress myocardial blood flow between LGE + and LGE- athletes. CMR characteristics according to presence of fibrosis are shown in Table 2.

**Table 2:** CMR characteristics of control group and athletes according to presence of fibrosis.

Characteristic	Healthy Control	Athlete LGE-	Athlete LGE +	*P* value Control vs LGE- athlete	*P* value Control versus LGE + athlete	*P* value LGE- versus LGE + athlete
LVEDV indexed to BSA (ml/m^2^)	82.9 ± 16.5	104.7 ± 15.1	108.0 ± 16.7	** < 0.001**	** < 0.001**	1.0
Rest LV EF (%)	63.6 ± 4.3	58.2 ± 6.2	58.2 ± 5.0	**0.001**	**0.001**	1.0
LV mass indexed to BSA (g/m^2^)	57.9 ± 8.7	75.7 ± 7.7	80.1 ± 11.5	** < 0.001**	** < 0.001**	0.31
RVEDV indexed to BSA (ml/m^2^)	90.3 ± 15.7	106.8 ± 17.2	109.7 ± 19.2	**0.003**	** < 0.001**	1.0
RV EF (%)	57.5 ± 7.0	56.8 ± 8.8	54.7 ± 7.9	1.0	0.64	1.0
Fibrosis on LGE (%)	4 (15)	0 (0)	24 (100)	–	–	–
Ischaemic fibrosis	1 (4)	0 (0)	0 (0)	–	–	–
Non-ischaemic fibrosis	3 (12)	0 (0)	24 (100)	–	–	–
Global native T1 (ms)	1268.6 ± 39.6	1250.6 ± 19.4	1248.0 ± 19.0	0.07	**0.03**	1.0
Extracellular volume fraction (%)	23.7 ± 1.6	23.1 ± 1.6	22.6 ± 1.9	0.58	0.07	0.95
Global T2 (ms)	41.7 ± 2.7	40.5 ± 1.7	40.1 ± 1.7	0.16	**0.04**	1.0
Stress Myocardial Blood Flow (ml/g/min)	1.9 ± 0.5	2.3 ± 0.7	2.2 ± 0.6	0.13	0.49	1.0
Rest Myocardial Blood Flow (ml/g/min)	0.6 ± 0.1	0.6 ± 0.1	0.6 ± 0.1	0.30	0.84	1.0
Myocardial Perfusion Reserve	3.1 ± 0.9	4.1 ± 1.2	3.9 ± 1.3	**0.015**	0.11	1.0
Fibrosis Volume (ml)	0.3 ± 0.9	0	2.6 ± 3.8	1.0	**0.001**	** < 0.001**
Extracellular matrix volume (ml)	26.2 ± 5.1	31.5 ± 4.0	31.7 ± 6.4	**0.001**	**0.001**	1.0
Cellular volume (ml)	83.6 ± 12.4	104.7 ± 9.9	107.8 ± 17.0	** < 0.001**	** < 0.001**	1.0

Values are mean ± standard deviation or n (%). *LGE*- Fibrosis absent; *LGE*+ Fibrosis present; *LVEDV* left ventricular end-diastolic volume; *BSA* body surface area; *LV EF* left ventricular ejection fraction; *LV* left ventricle; *RVEDV* right ventricular end-diastolic volume; *RV EF* right ventricular ejection fraction; *LGE* late gadolinium enhancement.

Significant values are in [bold].

In regard to competition history, there was no difference between LGE + and LGE – athletes in any of the parameters including number, distance or time spent in competitions (see supplementary material).

### ECG differences between athletes with and without fibrosis

There were no differences in any measured 12-lead ECG parameters between LGE + and LGE- athletes. On 24-h ECG monitoring, athletes with fibrosis had a greater burden of premature ventricular beats (PVBs) (0.3 ± 0.6 vs. 0.05 ± 0.2%, *p* = 0.03), with higher prevalence of ventricular couplets and triplets (33 vs. 8%, *p* = 0.02). ECG and 24-h monitor findings according to presence of fibrosis are shown in Table 3.

**Table 3:** Electrocardiogram and 24-h tape findings in athletes according to the presence of fibrosis.

	Athlete LGE-	Athlete LGE +	*P value*
PR interval (ms)	181.0 ± 33.5	180.4 ± 46.2	*0.96*
QRS duration (ms)	97.6 ± 7.9	97.0 ± 7.9	*0.78*
QTc (ms)	402.6 ± 84.0	419.3 ± 29.7	*0.36*
Early repolarisation (n, %)	9 (34.6)	12 (50.0)	*0.27*
T wave inversion (n, %)	0 (0)	2 (8.3)	*0.13*
Sokolow-Lyon criteria for LVH (n, %)	16 (61.5)	11 (45.8)	*0.24*
Minimum heart rate	45.1 ± 6.1	43.9 ± 5.4	*0.49*
Maximum heart rate	119.3 ± 26.9	120.3 ± 30.4	*0.90*
Proportion of time bradycardic (%)	6.5 ± 13.0	6.3 ± 11.2	*0.84*
Number of pauses	490.9 ± 1663.8	201.2 ± 513.2	*0.71*
Longest pause (s)	1.2 ± 1.1	1.1 ± 1.1	*0.75*
Number of PVBs	31.1 ± 96.1	201.5 ± 449.9	***0.01***
Percentage PVBs (%)	0.05 ± 0.2	0.3 ± 0.6	***0.03***
Patients with couplets/triplets (n, %)	2 (7.7)	8 (33.3)	***0.02***

Values are mean ± standard deviation, median (interquartile range) or n (%).

LGE- = Fibrosis absent; LGE +  = Fibrosis present; PVB = Premature ventricular beats.

Significant values are in [bold/italic].

### Myocardial characteristics in athletes with fibrosis

**Figure 2:**
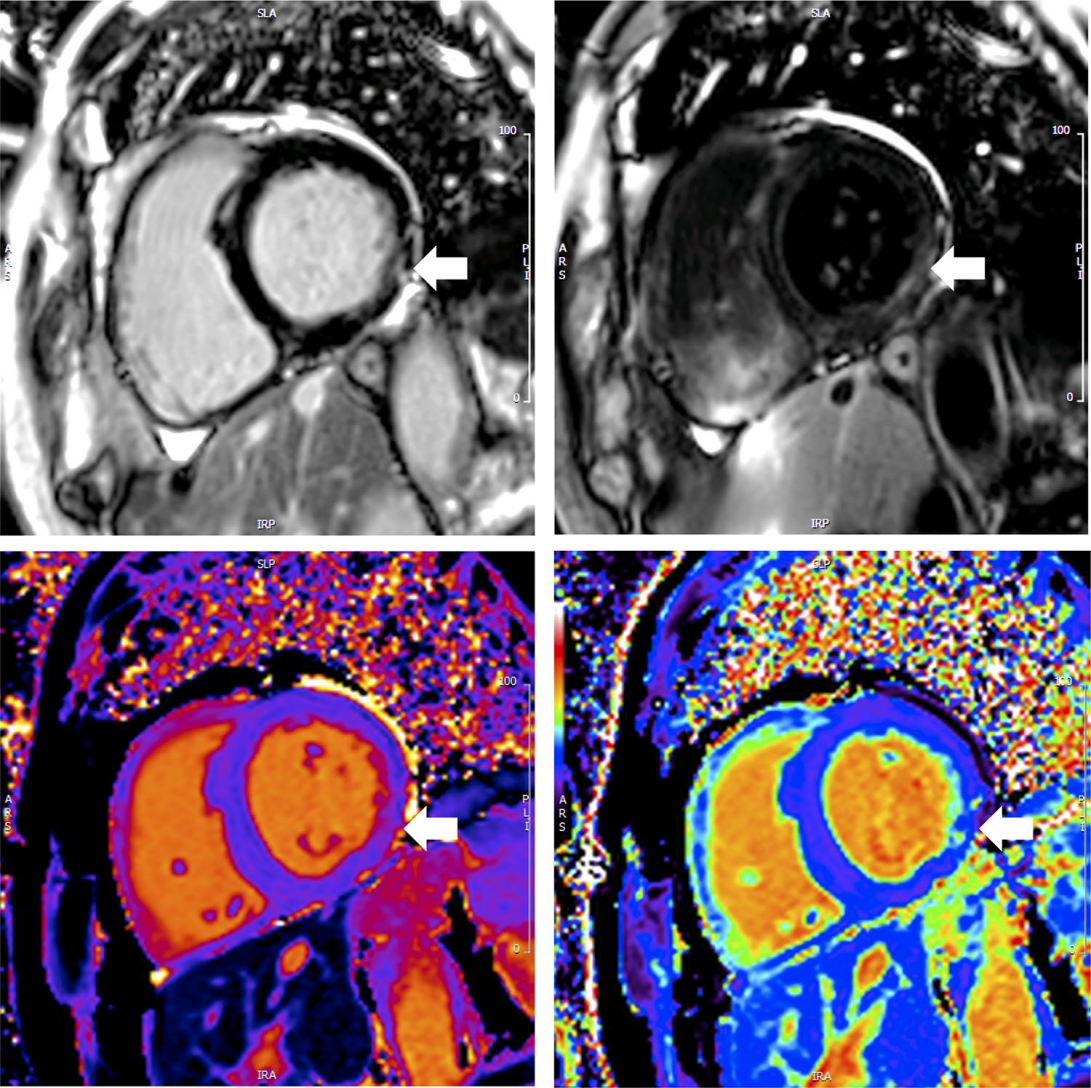
Fibrotic myocardial tissues characterisation. Multiparametric tissue characterisation of a single veteran athlete showing basal lateral mid wall fibrosis on bright blood LGE (upper left), dark blood LGE (upper right), native T1 mapping (lower left) and ECV mapping (lower right).

Fibrotic areas compared to healthy myocardium had higher native T1 (1367 ± 83 vs. 1249 ± 19 ms, *p* < 0.0001), ECV (32 ± 7 vs. 23 ± 2%, *p* < 0.0001) and T2 (44 ± 4 vs. 40 ± 2 ms, *p* < 0.0001) (Fig. 2 and Table 4). Rest myocardial blood flow was lower in fibrotic tissue (0.5 ± 0.1 vs. 0.6 ± 0.1 ml/g/min, *p* < 0.0001) but there was no difference in stress myocardial blood flow.

**Table 4:** Characteristics of fibrotic myocardium and remote regions from LGE + athletes.

	Fibrotic region	Remote region	*P value*
Native T1 (ms)	1366.9 ± 82.8	1249.4 ± 19.1	< *0.0001*
Extracellular volume fraction (%)	32.2 ± 6.6	22.9 ± 1.7	< *0.0001*
T2 time (ms)	44.3 ± 4.2	40.3 ± 1.7	< *0.0001*
Stress Myocardial Blood Flow (ml/g/min)	2.4 ± 1.0	2.2 ± 0.7	*0.63*
Rest Myocardial Blood Flow (ml/g/min)	0.5 ± 0.1	0.6 ± 0.1	< *0.0001*
Myocardial Perfusion Reserve	5.1 ± 1.8	4.0 ± 1.2	*0.009*

Values are mean ± standard deviation.

Significant values are in [italic].

## Discussion

**Figure 3:**
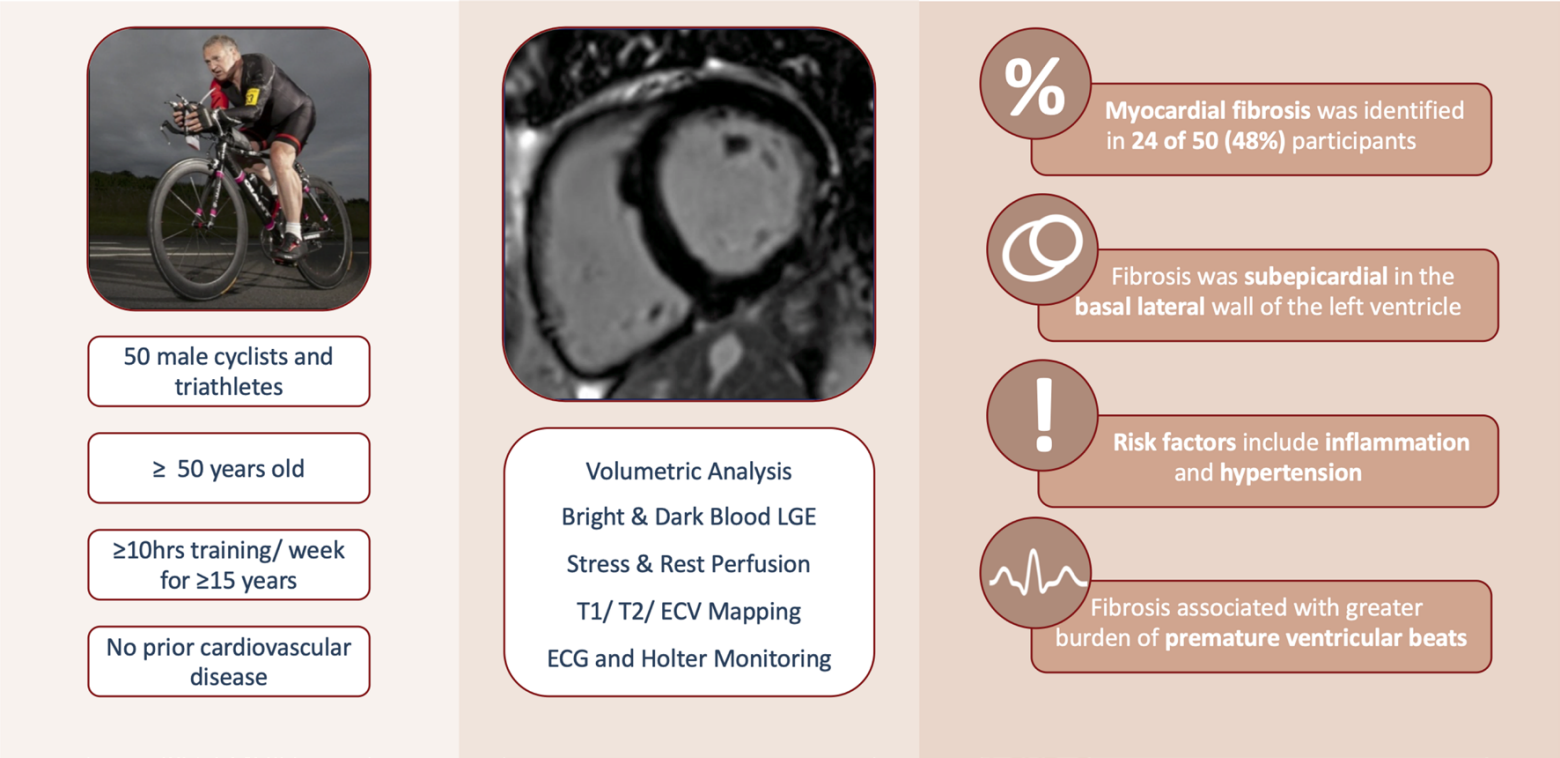
Non-Ischaemic Fibrosis in Veteran Endurance Athletes – Mechanisms and Association with Ventricular Ectopy. 50 veteran endurance athletes underwent cardiac MRI, 12-lead and 24-h ECG monitoring. Fibrosis was a common finding, identified in the basal lateral left ventricular wall in 48% of participants. Potential risk factors for myocardial fibrosis include inflammation and hypertension. The presence of fibrosis was associated with a greater burden of ventricular ectopics, including couplets and triplets. LGE = Late gadolinium enhancement. ECV = Extra-cellular volume.

We have demonstrated a 48% prevalence of myocardial fibrosis in veteran endurance athletes (compared with 15% in matched controls). There were no differences in any measured 12-lead ECG or CMR parameters between athletes with and without fibrosis. Athletes without fibrosis had lower blood pressure than controls, possibly implicating subclinical hypertension in the aetiology of fibrosis. Fibrotic areas of myocardium also had higher T2 values, suggesting a possible inflammatory mechanism. Furthermore, athletes with fibrosis had a greater burden of PVBs, with higher rates of ventricular couplets and triplets (Fig. 3).

### Prevalence of fibrosis in veteran endurance athletes

We identified myocardial fibrosis in 48% of veteran athletes in this study. In previous studies, myocardial fibrosis was found in 4–14% of athletes with age > 50^6–8^ and 9–17% in athletes age < 50 years^11,15^. Direct comparison with former studies is difficult due to different inclusion criteria and imaging techniques. There are several possible reasons why prevalence of fibrosis was increased in our study. Firstly, our inclusion criteria were relatively strict and we only included athletes with very high levels of competitive exercise. The stipulation of > 15 years of training at this level meant that athletes had a very high cumulative exposure of exercise. Secondly, we have only studied male athletes, who are known to have a higher prevalence of fibrosis than females^7,11^. Finally, we have used novel imaging techniques such as MOCO, DB LGE and ECV mapping that allowed us to identify relatively small areas of fibrosis with high diagnostic certainty.

The fibrosis identified in our study was exclusively mid-myocardial in the basal inferolateral segment. This pattern of fibrosis is the most commonly reported in other studies with similar inclusion criteria^6,7,11^. *Tahir *et al. reported prevalence of fibrosis in 17% of male triathletes training > 10 h per week^11^. Fibrosis was associated with exercise-induced hypertension and the cumulative distance raced. Other patterns of fibrosis reported in athletes includes RV insertion point fibrosis in 38%^16^ and ischaemic fibrosis in 5–7%^6,7^.

### Possible mechanisms for fibrosis

#### Myocarditis/inflammation

All fibrosis detected in our study was mid-myocardial, in the basal lateral wall of the left ventricle. Similar patterns have been reported in patients with prior myocarditis. In the acute phase of myocarditis, subepicardial hyperenhancement in the basal lateral segment is the most common finding on LGE imaging^17^. However, in the chronic phase, after six months, there are more varied patterns including mid-myocardial hyperenhancement similar to the pattern identified in our study^18^.

Although no participant reported a history of clinically diagnosed myocarditis, subclinical myocarditis in participants cannot be excluded. Furthermore, it has been speculated that exercising during a viral illness can exacerbate myocarditis. Cabinian et al. infected mice with coxsackievirus to induce myocarditis. Mice were assigned to groups for immunosuppressant therapy, exercise, both, or none. After 21 days, mortality was greatest in the exercise group^19^ suggesting exercise exacerbates myocardial damage.

In this present study, the T2 time was prolonged in regions of fibrosis, suggesting a possible inflammatory component. Initial screening studies of athletes affected by COVID-19 infection have suggested a variable prevalence of myocarditis^20,21^. Athletes in our study were scanned before the emergence of COVID-19 and future studies will be needed to establish if the pandemic leads to increased levels of long-term myocardial fibrosis in athletes.

#### Subclinical hypertension

Athletes with myocardial fibrosis in our study had blood pressure which was similar to controls, whereas those without fibrosis had significantly lower blood pressure. Whilst rest blood pressures in our study were well within the normal range, it is well recognised that during sport there can be marked increases in blood pressure (particularly systolic). In marathon runners, a positive correlation was found between blood pressure and LV mass, as well as association between LV mass and coronary artery calcium scores^22^. Tahir et al. previously noted that young athletes with non-ischaemic fibrosis had an increased incidence of hypertension during exercise, and higher LV mass^11^. In this present study, there was no difference in LV mass between athletes with and without fibrosis. The pattern of fibrosis seen in arterial hypertension is predominantly mid-myocardial, most commonly affecting mid and basal inferior and inferolateral segments^23^. It is possible that exercise-induced hypertension may be a contributor to the findings of our study.

#### Ischaemic heart disease

Previous studies have demonstrated the presence of both coronary^7,24^ and extra-coronary^25^ atherosclerosis in veteran athletes, even in the absence of angina. Some studies have also reported increased prevalence of ischaemic fibrosis^6,7^. In addition, both presence of fibrosis and increasing coronary artery calcium scores were associated with higher coronary events^26^. It is possible that occult coronary artery disease was a contributing factor to the fibrosis seen in our study, although this is a less likely aetiology as there was no evidence of ischaemic subendocardial fibrosis or inducible regional ischaemia on stress perfusion imaging.

#### Chronic volume loading

A similar pattern of basal lateral fibrosis has been reported in patients with chronic mitral regurgitation secondary to primary mitral valve disease. Although the mechanism is physiological rather than pathological dilatation, long term participation in endurance sport may similarly lead to chronic volume loading of the left ventricle, with subsequent fibrosis from mechanical remodeling. In these patients with chronic mitral regurgitation basal lateral midwall fibrosis appears to be associated with ventricular arrhythmia^27,28^.

### Association between fibrosis and ventricular arrhythmia

This study has demonstrated a greater percentage of PVBs, including couplets and triplets, on 24-h ECG monitoring of veteran endurance athletes with myocardial fibrosis compared to athletes without fibrosis. The presence of non-ischaemic fibrosis is associated with adverse outcomes including ventricular arrhythmia in a variety of conditions including dilated, hypertrophic and arrhythmogenic cardiomyopathies^9^. In non-athletic patients the presence and burden of ventricular ectopy are markers of incident heart failure and mortality^29^. The relationship between PVBs and outcomes is less clear in athletes. According to the 2015 recommendations of the American Heart Association and the American College of Cardiology, athletes with PVBs or couplets should be considered for imaging including CMR to identify cardiomyopathies, anomalous coronary artery origins, and subclinical myocarditis. In the absence of structural heart disease athletes should be considered eligible for all competitive sports but the recommended intensity of exercise is to remain under the threshold for the occurrence of arrhythmia-related symptoms such as presyncope, syncope or dyspnoea^30^.

Prospective evidence that athletic myocardial fibrosis leads to ventricular arrhythmia is lacking. However, there are preclinical and retrospective data from symptomatic athletes that suggest myocardial fibrosis may be a substrate for ventricular arrhythmia. Rats forced to run an hour a day for up to 16 weeks develop physiological myocardial hypertrophy and dilation, alongside fibrosis predominantly affecting the atria and right ventricles^31^. Ventricular tachycardia could be induced in 5 of 12 exercise rats (42%) and only 1 of 16 sedentary rats (6%; *P* = 0.05). Interestingly there was reversal of fibrosis after 8 weeks of exercise cessation. Only rats that could maintain the very intense exercise regime were studied (selection bias) and it is not clear whether the mechanisms of fibrosis in rats undergoing short-term intense exercise is the same as in humans after a lifetime of endurance training.

In two publications of 27 and 37 athletes suffering ventricular arrhythmia, non-ischaemic fibrosis was found by LGE CMR in 22%^32,33^. Zorzi et al. reported the most common pattern of fibrosis was subepicardial/midmyocardial affecting the basal lateral wall in 77% of those with fibrosis^33^. Subjects for these studies were identified retrospectively after they presented with ventricular arrhythmia making findings vulnerable to selection bias. Furthermore, it is unclear as to what extent the fibrosis was caused by athletic training, co-existent cardiomyopathy, or a combination of both.

### Limitations

This small sample of veteran athletes underwent a limited duration of ECG monitoring, the duration of which was insufficient to capture malignant arrhythmias. Control subjects did not undergo ECG monitoring. Although we identified differences in PVBs rates, this may not necessarily reflect arrhythmic event rates in the future. Hence, long term studies reporting such events in such cohorts are required.

Our findings in relation to competition history are based on self-reported data, which relies on the accuracy of recall of the athletes. The association with objective levels of fitness cannot be ruled out as participants did not undergo cardiopulmonary exercise tests. We also only recruited cyclists and triathletes; hence these findings may not be representative of other sports.

## Conclusions

In life-long veteran endurance athletes, myocardial fibrosis is a common finding. Possible mechanisms for fibrosis include inflammation and elevated arterial blood pressure. Presence of myocardial fibrosis is associated with an increased burden of ventricular ectopy. Further studies are needed to establish whether fibrosis increases risk of malignant arrhythmic events.

### Supplementary Information

Supplementary Tables.

## Data Availability

The datasets generated and analysed during the current study are available from the corresponding author on reasonable request.
