# Supplementary material for: Identification of non-ischaemic fibrosis in male veteran endurance athletes, mechanisms and association with premature ventricular beats
Source: Sci Rep. 2023 Sep 5;13:14640. doi: 10.1038/s41598-023-40252-z (PMC10480152; doi:10.1038/s41598-023-40252-z)
Supplement: Supplementary file 1 — Supplementary Tables. [file 41598_2023_40252_MOESM1_ESM.pdf]

Table 1: Cycling competition history according to presence of fibrosis.

	Cyclist LGE-	Cyclist LGE+	*P value*
**N**	20	21	
**Active Years**	24.4 ± 11.3	28.4 ± 14.5	*0.34*
**Training per week (hrs)**	11.5 ± 1.8	11.5 ± 2.1	*0.91*
**Total no. Competitions**	622.5 ± 587.7	702.1 ± 567.4	*0.67*
**Competitions per year**	22.8 ± 13.4	31.3 ± 27.7	*0.22*
**Total distance (miles)**	22 853.7 ± 33 546.7	33 595.0 ± 38 063.1	*0.35*
**Average distance per race (miles)**	40.4 ± 59.9	49.0 ± 43.3	*0.61*
**Total time (hrs)**	1194.1 ± 1456.3	1633.2 ± 1610.2	*0.37*

Values are mean ± standard deviation.

LGE- = Fibrosis absent; LGE+ = Fibrosis present.

Table 2: Triathlon competition history according to presence of fibrosis.

	Triathlete LGE-	Triathlete LGE+	*P value*
**N**	6	3	
**Active Years**	25.5 ± 9.4	23.0 ± 13.0	*0.75*
**Training per week (hrs)**	11.3 ± 1.7	10.7 ± 1.2	*0.61*
**Total no. Competitions**	215.3 ± 195.7	81.7 ± 89.8	*0.31*
**Competitions per year**	7.5 ± 5.3	3.0 ± 2.6	*0.22*
**Total swimming distance (km)**	247.4 ± 175.6	102.3 ± 99.6	*0.23*
**Total cycling distance (km)**	7343.3 ± 4596.2	3073.3 ± 2731.0	*0.19*
**Total running distance (km)**	1807.3 ± 1149.6	759.3 ± 672.0	*0.20*
**Total distance (km)**	9398.0 ± 5918.1	3935.0 ± 3500.6	*0.19*
**Total time (hrs)**	465.7 ± 270.6	185.7 ± 153.7	*0.15*
**No of sprint distances**	103.0 ± 134.5	37.3 ± 46.5	*0.45*
**No of Olympic distances**	316.0 ±588.9	35.7 ± 47.5	*0.45*
**No of middle distances**	11.0 ± 8.7	2.7 ± 3.1	*0.16*
**No of iron man distances**	4.0 ± 7.5	3.3 ± 4.9	*0.90*

Values are mean ± standard deviation.

LGE- = Fibrosis absent; LGE+ = Fibrosis present.
